# P2X1 receptor mobility and trafficking; regulation by receptor insertion and activation

**DOI:** 10.1111/j.1471-4159.2010.06730.x

**Published:** 2010-06

**Authors:** Ulyana Lalo, Rebecca C Allsopp, Martyn P Mahaut-Smith, Richard J Evans

**Affiliations:** Department of Cell Physiology & Pharmacology, Henry Wellcome Building, University of LeicesterLeicester, UK

**Keywords:** ATP, FRAP, GFP, P2X, regulation, trafficking

## Abstract

P2X1 receptors for ATP contribute to signalling in a variety of cell types and following stimulation undergo rapid desensitisation (within 1 s), and require ∼5 min to recover. In HEK293 cells P2X1 receptors C-terminally tagged with enhanced green fluorescent protein (P2X1-eGFP) were predominantly expressed at the cell surface. Following > 90% photo-bleaching of P2X1-eGFP within a 6 μm^2^ circle at the cell surface fluorescence recovery after photo-bleaching (FRAP) was fit with a time constant of ∼60 s and recovered to ∼75% of pre-bleach levels. Following activation of the P2X1 receptor with α,β-methylene ATP the associated calcium influx doubled the FRAP recovery rate. The protein synthesis inhibitor cycloheximide had only a small effect on repeated FRAP and indicated a limited contribution of new P2X1 receptors to the FRAP. Inhibition of trafficking with brefeldin A reduced recovery and this effect could be reversed following receptor activation. In contrast, the dynamin inhibitor dynasore had no effect on FRAP under unstimulated conditions but reduced the level of recovery following agonist stimulation. In functional studies both brefeldin A and dynasore increased the recovery time from desensitisation. Taken together these studies demonstrate for the first time an important role of receptor recycling on P2X1 receptor responsiveness.

P2X receptors for ATP comprise a distinct family of ligand-gated cation channels with two transmembrane segments, and a large extracellular ligand binding loop with intracellular amino and carboxy termini (for reviews of properties see Egan *et al.* 2004; Burnstock 2006; Khakh and North 2006; Roberts *et al.* 2006; Surprenant and North 2009). There are seven mammalian P2X receptor subunits (P2X1–7) which can form homo- and hetero-trimeric receptors with a range of properties (North 2002). P2X1 receptors play important roles in neurogenic smooth muscle contraction (Mulryan *et al.* 2000; Vial and Evans 2000, 2002), platelet activation (Hechler *et al.* 2003; Mahaut-Smith *et al.* 2004), as well as neuronal (Calvert and Evans 2004; Watano *et al.* 2004) and glial cell responses (Lalo *et al.* 2008). A characteristic feature of P2X1 receptors is that they show rapid receptor desensitisation (time constant ∼250 ms), and ∼ 5 min is required for recovery following agonist washout (Valera *et al.* 1994; Lewis and Evans 2000). The mechanisms underlying recovery from desensitisation remain unclear. The run-down of P2X1 receptor currents in whole cell recordings, but not in permeabilised patches, suggests that intracellular factors are involved (Lewis and Evans 2000). In addition, P2X1 receptors have been reported to internalise following activation (Dutton *et al.* 2000; Li *et al.* 2000; Ennion and Evans 2001) that may also contribute to the desensitisation process. P2X1 receptors can also be potentiated by activation of Gαq G protein coupled receptors (GPCRs) and phorbol esters, e.g. phorbol-12-myristate-13-acetate (PMA) (Vial *et al.* 2004; Ase *et al.* 2005; Wen and Evans 2009), however the underlying mechanism of this cross-sensitisation and the extent to which the P2X1 receptor can be regulated by other classes of GPCRs is unknown.

Trafficking of receptors can play an important role in the regulation of responsiveness. A conserved YXXXK membrane targeting sequence in the intracellular C-terminal domain is important for delivery of P2X receptors to the cell surface and disruption of this motif reduced ATP-evoked currents by > 95% (Chaumont *et al.* 2004). P2X4 receptors show constitutive internalisation through a dynamin dependent pathway (Bobanovic *et al.* 2002) and sequestration to lysosomes (Qureshi *et al.* 2007). P2X3 receptors also show constitutive receptor internalisation however agonist stimulation leads to transient up-regulation of surface receptor expression and subsequent acceleration of internalization (Vacca *et al.* 2009). To date, however it is unclear what role trafficking or membrane diffusion plays in the characteristic rapid desensitisation and slow recovery process exhibited by P2X1 receptors. Fluorescent recovery after photo-bleaching (FRAP) of green fluorescent protein-tagged receptors and ion channels has been used to track channel movement (e.g. O’Connell *et al.* 2006) and gives a real time measure of mobility. For example, FRAP has been used to monitor P2X2-enhanced green fluorescent protein (eGFP) dynamics (Chaumont *et al.* 2008) and receptor activation leads to receptor redistribution in hippocampal neurons (Khakh *et al.* 2001).

The recovery of fluorescence following photo-bleaching can result from the trafficking of new receptors to the cell surface, receptor recycling, and/or lateral diffusion of receptors from adjacent stretches of the plasma membrane. For example the trafficking of newly synthesised receptors regulates P2X3 receptor surface expression (Vacca *et al.* 2009) and recycling plays a role in epithelial sodium channel expression (Butterworth *et al.* 2005). In the present study we have used FRAP to determine the mobility and trafficking of P2X1 receptors with eGFP fused to the C-terminus (P2X1-eGFP). We show that P2X1 receptors exhibit both constitutive and agonist induced recycling that contribute to recovery from desensitisation. Overall the results show that recycling plays an important role in the regulation of P2X1 receptor responsiveness.

## Methods

### Generation of enhanced green fluorescent protein-tagged P2X receptors

Oligonucleotides were designed to add the restriction sites *Hind*III and *Bam*H1 to the N- and C-terminus respectively of the human P2X1 receptor (Vial and Evans 2005). Similarly *Hind*III and *Eco*R1 were added to the N- and C-terminus respectively of the human P2X2 receptor. Using these sites P2X receptors were sub-cloned into the multiple cloning site of the peGFP-N1 vector (Clontech, Basingstoke, UK.), making sure that it was in frame with the eGFP coding sequences with no intervening in-frame stop codons (stop codon point mutated to tryptophan). The sequence integrity of all constructs was verified by DNA sequencing using Leicester University PNACL services.

### Cell culture and transfection of HEK293 cells

Native HEK293 were maintained in minimal essential medium with Earle’s Salts (with GlutaMAX™ I, Invitrogen, Carlsbad, CA, USA) supplemented with 10% foetal bovine serum and 1% non-essential amino acids (Invitrogen) at 37°C in a humidified atmosphere of 5% CO_2_ and 95% air. A monolayer of native cells at 80–90% confluence in a 24-well culture dish was transiently transfected using 0.5 μg DNA (human P2X1-eGFP, human P2X2-eGFP or eGFP) and 1 μL lipofectamine 2000 (Invitrogen) in 500 μL of serum-free Opti-MEM1. After 24-h incubation, cells were plated onto 25 mm No 0 or 13 mm No 1 coverslips for FRAP and electrophysiological experiments and left to grow in Dulbecco’s modified Eagle’s medium cell culture medium. Cells were subjected to experiments 24–48 h after transfection.

### FRAP measurements

Confocal fluorescence measurements were made on an Olympus inverted microscope with a confocal laser scanning module **(**Olympus FluoView1000, Southend on Sea, Essex, UK). Cells were observed with a 60× oil immersion objective lens (UPLSAPO 60XO, NA 1.35, Southend on Sea, Essex, UK). eGFP fluorescence was monitored using 488 nm excitation and 500–600 nm emission. Images were collected at a rate of 0.5 Hz. Each FRAP experiment started with collection of 30 baseline control images of the cell followed by a 1-s duration photo-bleach of the region of interest using high intensity 488 nm illumination sufficient to photobleach > 90% of the eGFP fluorescence. Typically, 100 images were then recorded at normal laser intensity after bleaching. A 40 mW multi-line argon laser was used for both imaging and photobleach, at outputs of 0.8% and 20% respectively of the 488 nm line. Average fluorescence intensities of bleached, non-bleached, and background regions were recorded for each time point. Fluorescence signals were background-subtracted, corrected for fading of fluorescence during experiment and expressed as *F/F*_i_ ratios to normalise fluorescence levels (*F*) against starting fluorescence (*F*_i_). Fluorescence intensities of the region of interest were obtained using FluoView software and plotted. In experiments with cycloheximide (10 μg/mL for 2 h), brefeldin A (10 μg/mL for 2 h) or dynasore (80 μM for 1 h) cells were pre-treated in drug-containing solution before the experiment. Rapid drug application from a U-tube perfusion system caused cellular movement incompatible with FRAP measurements, thus for studies of P2X1 receptor activation α,β-methylene ATP (α,β-meATP, 1 μM) was applied directly to the cells just before the FRAP imaging.

### Electrophysiological recordings and solutions

Permeabilized patch voltage-clamp recordings were made from HEK293 cells using a Axopatch 200B amplifier (Axon Instruments, Union City, CA, USA). Membrane currents were recorded at a holding potential of −70 mV (corrected for tip potential). Data were low pass-filtered at 1 kHz, digitized at a sampling interval of 200 μs and acquired using a Digidata 1200 analogue-to-digital converter with pClamp 9.2 acquisition software (Molecular Devices, Palo Alto, CA, USA). Microelectrodes were filled with internal solution composed of (in mM) K gluconate, 140; EGTA, 10; HEPES, 10; NaCl, 5 (pH 7.3, adjusted with KOH) and had resistances in the range of 2–6 MΩ. For perforated patch pipette recordings amphotericin B was added to the internal solution at a final concentration of 200 μg/mL. The bath was continuously perfused with extracellular solution containing (in mM) NaCl, 150; KCl, 2.5; CaCl_2_, 2.5; MgCl_2_, 1; HEPES, 10; glucose, 10 (pH 7.3, adjusted with NaOH). α,β-meATP was applied with a U-tube perfusion system. Repeated applications of agonist were separated by 5 min in order to allow recovery from receptor desensitization. In experiments with brefeldin A (10 μg/mL for 2 h) or dynasore (80 μM for 1 h) cells were pre-treated in drug-containing solution before the experiment. During the permeabilized patch recording PMA (100 nM), forskolin (10 μM) or somatostatin (500 nM) were added to extracellular solution. Control for treated cells was done every day therefore the comparisons between non-treated and treated cells were made between the cells from the same batch. All chemicals were purchased from Sigma (Poole, Dorset, U.K.).

### Data analysis

**Fig. 1:**
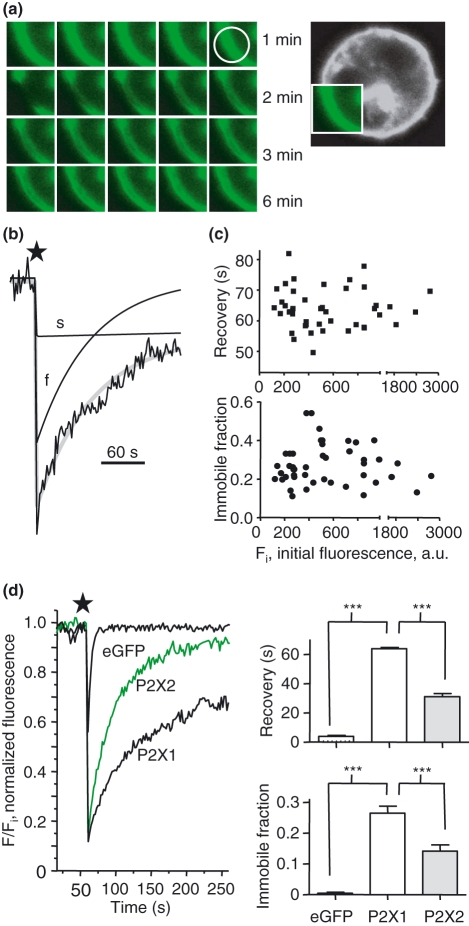
Characterization of P2X1 receptor mobility by FRAP. (a) HEK293 cells were transfected with P2X1-eGFP DNA. Right hand panel shows the whole cell and the square is shown at higher power in a time series of fluorescent images. Images were obtained with the laser scanning confocal microscope before (first minute) and after photo-bleaching of the region indicated by the circle. (b) Example of P2X1 receptor FRAP fit by two components. The black jagged line represents the experimental curve. The lines labelled f and s represent the fast and slow/sustained components of the recovery, the gray line represents the sum of the components. Photobleach is indicated by the star. (c) Distribution of recovery time constants and immobile fractions of individual cells transfected with P2X1-eGFP DNA is independent of the level of initial fluorescence (*n* = 67). (d) Representative FRAP traces and summary data of recovery time constants and immobile fractions for cells transfected with P2X1-eGFP, P2X2-eGFP and eGFP DNA (*n* = 67, 18 and 8 respectively) indicate that FRAP parameters are subunit specific. Star indicates photo-bleach. ***p < 0.001.

Data were analyzed with CLAMPFIT (Molecular Devices) or ORIGIN 6.0 (Microcal Software, Northampton, MA, USA). The kinetics of recovery were analysed by fitting the curve of fluorescence intensity with model curves with monoexponential rise and decay phases (Fig. 1b). The time constant of recovery (τ) was determined as the time constant of fast component and optimized by the gradient method to minimize the mean square error (Pankratov and Krishtal 2003). Data in the text and graphs are shown as mean ± SEM from *n* determinations as indicated and analyzed using the unpaired Student’s *t*-test and *p* < 0.05 was considered significant.

## Results

### Characterisation of P2X1 FRAP

P2X1-eGFP fluorescence was observed predominantly at the plasma membrane and associated with intracellular structures of cells transiently transfected with P2X1-eGFP DNA (Fig. 1a). The addition of the eGFP tag to the C-terminus of the P2X1 receptor had no effect on agonist sensitivity (α,β-meATP pEC_50_ 5.48 ± 0.10 and 5.53 ± 0.07, Hill slope, 2.1 ± 0.4 and 1.8 ± 0.2, current density 234 ± 38 and 245 ± 44 pA/pF, for P2X1 wild type and P2X1-eGFP respectively), or desensitisation (time constant for decay 242 ± 25 and 238 ± 18 ms respectively for P2X1 wild type and P2X1-eGFP) and was therefore used in FRAP studies to investigate receptor mobility. For photo-bleaching, a 6 μm^2^ circle was selected at the cell surface and illuminated with high intensity 488 nm light for 1 s (see methods for further details) sufficient to cause an ∼ 90% decrease in fluorescence within the selected region. There was ∼ 75% recovery of fluorescence to the bleached area within 5–6 min (Fig. 1). The recovery was fit with a fast single exponent (f) with a time constant (τ) of 63.1 ± 1.5 s (*n* = 67) and a sustained bleached (s) component (Fig. 1b). There was a linear correlation between the recovery rate and the area of photo-bleaching within the range of 3–9 μm^2^ (data not shown). The recovery could result from a combination of lateral diffusion of P2X1-eGFP tagged receptors from the adjacent membrane as well as the insertion of either new and/or recycling receptors. The sustained bleached fraction (0.26 ± 0.01, *n* = 67) corresponds to receptors that were bleached and remain within the area of interest (Axelrod *et al.* 1976) and will subsequently be referred to as the immobile fraction (Fig. 1c). The recovery time and immobile fraction were both independent of the level of expression of eGFP-tagged receptors (Fig. 1c).

For comparison we also determined FRAP for cytosolic eGFP and surface P2X2-eGFP. When eGFP alone was expressed in HEK293 cells, recovery from photo-bleaching was very fast (time-constant 4.01 ± 0.68 s, *n* = 6) and complete (Fig. 1d). C-terminally eGFP tagged P2X2 receptors showed FRAP with a similar single exponential recovery and sustained bleach however recovery was twice as fast as the P2X1 receptor (31.2 ± 2.1 s, *n* = 20), and the fraction of immobile P2X2-eGFP receptors was half that for P2X1-eGFP (0.14 ± 0.019 and 0.26 ± 0.01 respectively, *p* = 0.001). These results demonstrate that P2X receptors show subunit dependent mobility in the membrane. However in the present study we have focused our attention on the mechanisms involved in the regulation of P2X1 receptor FRAP.

### Effects of P2X1 receptor activation on mobility

**Fig. 2:**
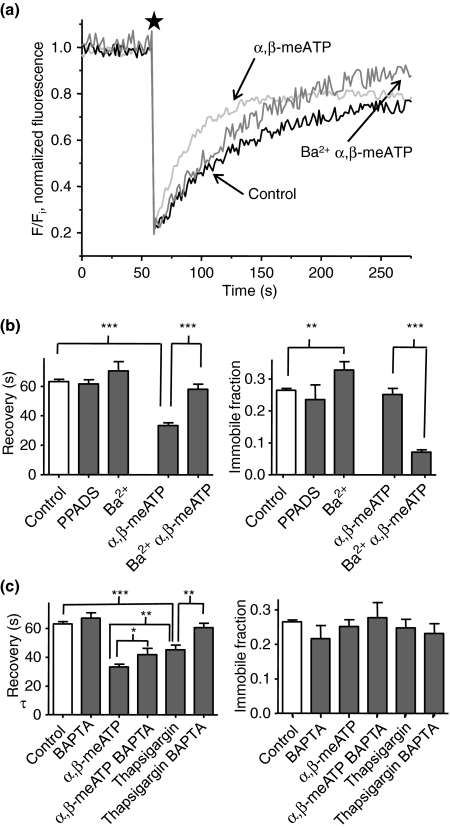
Speeding of FRAP following P2X1-eGFP receptor activation is dependent on localized Ca^2+^ microdomains. (a) Representative FRAP traces in control conditions and in the presence of 1 μM α,β-meATP with calcium or barium in the extracellular solution. (b) FRAP time constants and immobile fractions in control conditions were not affected in the presence of PPADS, but activation of P2X1 receptors by α,β-meATP increased the rate of recovery about twofold (*n* = 17, *p* < 0.0001). This effect of α,β-meATP was abolished when the calcium in the extracellular solution was replaced with barium (*n* = 10, *p* < 0.0001). (c) Summary of the effects of BAPTA (50 μM BAPTA-AM for 1 h), α,β-meATP, and thapsigargin on FRAP rate and immobile fraction. These indicate that direct local calcium influx plays a role in the speeding of FRAP following P2X receptor activation. Star indicates photo-bleach. **p < 0.01, ***p < 0.001.

P2X1 receptors show profound desensitisation on agonist stimulation. To determine whether activation of the P2X1 receptor influences mobility we treated HEK293 cells expressing the P2X1-eGFP receptor with 1 μM α,β-meATP. Following P2X1 receptor activation the rate of FRAP was doubled (32.5 ± 2.1 s, *n* = 17), while the immobile fraction of receptors was unaffected (Fig. 2). The P2X1 receptor antagonist phosphate-6-azophenyl-2′,4′-disulfonate (PPADS) (10 μM), applied in the absence of α,β-meATP, had no effect on the rate of recovery or the immobile fraction (61.4 ± 2.9 s and 0.23 ± 0.04 s respectively, *n* = 6) demonstrating that there was no basal effect of endogenous release of ATP from the cultured cells. P2X1 receptors show high calcium permeability and ∼ 12% of the current flowing under normal physiological conditions is carried by calcium (Egan and Khakh 2004). To test whether receptor activation and/or calcium influx mediated the speeding in FRAP, we replaced the calcium in the extracellular solution with barium. In the absence of agonist barium substitution had no effect on the speed of recovery but caused a small increase in the immobile fraction (70.4 ± 6.2 s and 0.32 ± 0.03 s respectively, *n* = 5, Fig. 2). Replacement of calcium with barium prevented the acceleration of FRAP by α,β-meATP suggesting that an increase in intracellular calcium plays a key role in regulating the speed of movement of the receptors (Fig. 2). Interestingly, α,β-meATP applied with barium also resulted in a small ∼ 15% reduction of the immobile fraction (Fig. 2), however the underlying reasons for this effect remains to be determined.

To explore further whether calcium influx alone, or the combined activation of the P2X receptor, coupled with the rise in intracellular calcium, was responsible for the accelerated FRAP, we raised intracellular calcium independently with thapsigargin (Ribeiro *et al.* 2000). A speeding in the recovery from photo-bleaching, with no effect on the immobile fraction was also seen following treatment with thapsigargin (10 min with 1 μM) (time constant for FRAP 45 ± 3.2 s, *n* = 9) indicating that an increase in calcium alone, and not co-incident activation of the channel was sufficient to speed FRAP (Fig. 2c). 1,2-Bis(2-aminophenoxy) ethane-N,N,N′,N′-tetraacetic acid tetrakis(acetoxymethyl ester) (BAPTA-AM) treatment (50 μM, 1 h at 37°C) was used to buffer rises in intracellular calcium and abolished the increase in recovery from photo-bleaching in response to thapsigargin but only had a small reduction on α,β-meATP induced speeding (Fig. 2c). BAPTA will be more effective as a buffer of the slow thapsigargin evoked calcium increases through store release compared to the rapid P2X1 receptor evoked influx. The rapid, and localised microdomain of calcium following P2X1 receptor activation, even in the presence of BAPTA, was sufficient to speed the recovery from photo-bleaching and suggests that the appreciable calcium permeability of the P2X1 receptor contributes locally at the membrane to receptor mobility.

### Do newly synthesised P2X receptors contribute to FRAP?

**Fig. 3:**
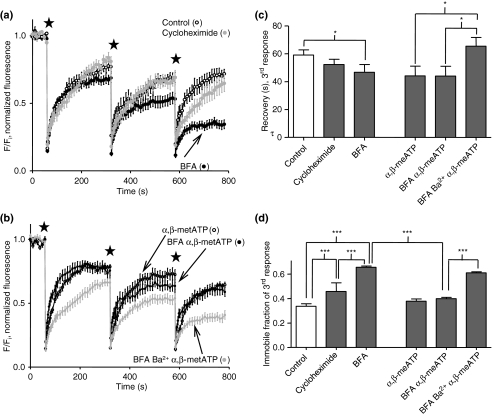
Turnover of P2X1-eGFP receptors is brefeldin A (BFA) sensitive. (a) Averaged FRAP traces for triple serial photo-bleaching (bleach indicated by star) of the same area for non-treated (control) cells, cells treated with cylcloheximide (10 μg/mL for 2 h) or BFA (10 μg/mL for 2 h). (b) Averaged FRAP traces for triple serial photo-bleaching of the same area for P2X1-eGFP receptors. When P2X1 receptors were activated with 1 μM α,β-meATP BFA had no effect on serial photobleaching, however when barium replaced calcium in the external solution BFA again reduced FRAP. (c) Summary of the effects of cycloheximide, BFA, α,β-meATP and barium on the time-course of recovery to the third photobleach. Star indicates photo-bleach. (d) Summary of the effects of cycloheximide, BFA, α,β-meATP and barium on the immobile fraction at the end of three rounds of photobleaching. Star indicates photo-bleach. *p < 0.05, ***p < 0.001.

The recovery of fluorescence following photo-bleaching could result from the migration of receptors already present at the membrane or trafficking of new receptors to the cell surface. Cells were given three rounds of photo-bleach to characterise further recovery from photo-bleaching. The immobile fraction was initially 0.26 ± 0.04 and increased on a second photo-bleach to 0.33 ± 0.09 (*p* < 0.005) and was then stable on a third bleach at 0.30 ± 0.01 (*n* = 16). This stabilisation of the immobile fraction could result from a fraction of the P2X1 receptors being retained within cell surface microdomains as shown for Kv2.1 potassium channels (O’Connell *et al.* 2006) and thus unable to move out of the photo-bleach area. Cells were incubated with cycloheximide (10 μg/mL for 2 h), an inhibitor of protein synthesis, to determine the contribution of trafficking of new P2X1-eGFP receptors to the cell surface. Cycloheximide had no effect on the rate of FRAP. Initially there was no effect on the immobile fraction to the first photo-bleach, however following three rounds of photo-bleaching there was a ∼ 12% increase in the immobile fraction (Fig. 3a). This suggests that trafficking of new P2X1 receptors to the cell surface plays only a small role in receptor turnover in the short term (and confirms a recent study looking at surface expression of P2X1 receptors (Vacca *et al.* 2009).

### Does receptor recycling contribute to P2X1 receptors FRAP?

The limited contribution of newly synthesised P2X1 receptors to FRAP suggests that recovery results predominantly from lateral diffusion of receptors already at the cell surface and/or the insertion of recycling receptors. To determine the relative contribution of insertion of recycling P2X1 receptors we have used the cell-permeant inhibitors of vesicle trafficking brefeldin A (BFA) (Hunziker *et al.* 1991; Chardin and McCormick 1999) and the dynamin inhibitor dynasore (Macia *et al.* 2006; Thompson and McNiven 2006).

Brefeldin A treatment (10 μg/mL for 2 h, 37°C) speeded the recovery rate (33 ± 5.3 s, *n* = 10) and resulted in a progressive increase in the immobile fraction following repeated photo-bleaching (Fig. 3a). At the end of the third recovery period the immobile fraction had increased from 0.337 ± 0.007 to 0.656 ± 0.003 (*n* = 10 and *p* < 0.001) (Fig. 3). In contrast BFA had no effect on the rate of recovery or the P2X2 receptor FRAP immobile fraction at the end of the third photo-bleach (0.27 ± 0.01, *n* = 6 and 0.24 ± 0.007, *n* = 5 for control and BFA treated respectively, *p* = 0.025). This demonstrates that the effects of BFA on the P2X1 receptor are not the result of non-specific effects such as general cellular disruption. The BFA effect on the P2X1 receptor immobile fraction suggests that constitutive vesicular trafficking (recycling) of the receptor makes a significant contribution to the expression of P2X1 receptors at the cell surface. Interestingly, following P2X1 receptor activation with α,β-meATP, BFA had no effect on the immobile fraction in response to repeated photo-bleaching (Fig. 3). This rescue of recovery was abolished when barium replaced calcium in the extracellular solution (Fig. 3). These results suggest that in addition to the BFA sensitive constitutive component there is a calcium-sensitive (BFA-insensitive) pathway triggered by P2X1 receptor activation that contributes to receptor trafficking.

**Fig. 4:**
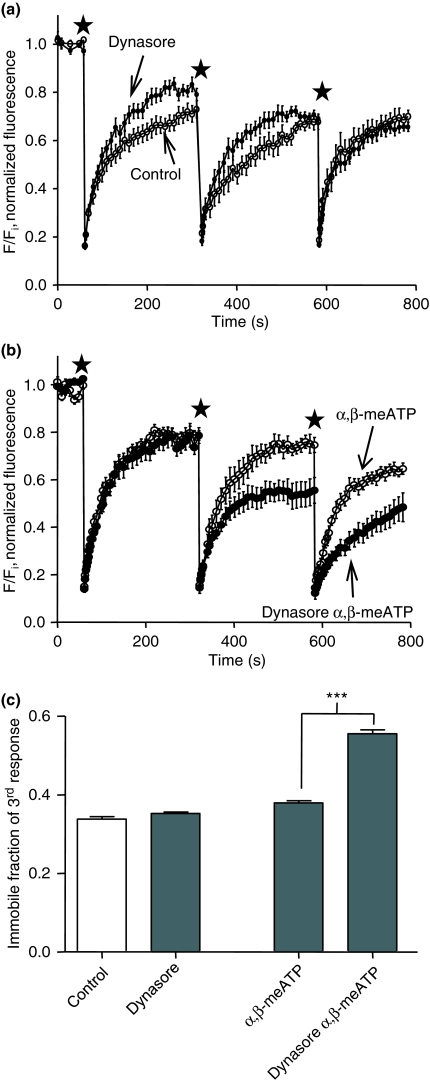
Agonist and dynamin dependent P2X1 receptor internalization/recycling. (a) Averaged FRAP traces for triple serial photo-bleaching (bleach indicated by star) of the same area for non-treated (control) cells and cells treated with dynasore (80 μM for 1 h). (b) Averaged FRAP traces for triple serial photo-bleaching of the same ROI when P2X1-eGFP receptors were activated by 1 μM α,β-meATP in control or dynasore treated cells. (c) Summary of the effects of dynasore on the immobile fraction at the end of the third photo-bleach. Star indicates photo-bleach. ***p < 0.001.

Agonist-stimulated receptor recycling can occur through a dynamin dependent pathway. Dynasore is a cell permeable inhibitor of dynamin 1 and dynamin 2 GTPase activity and has been used to block the formation of clathrin coated vesicles (for mini review see Thompson and McNiven 2006). Dynasore (80 μM, 1 h, 37°C) had no effect on P2X1 receptor FRAP under resting conditions with no agonist present (Fig. 4). However following P2X1 receptor activation with α,β-meATP there was an increase in the immobile fraction in successive rounds of photo-bleaching with a ∼ 20% increase at the end of the third photo-bleach (Fig. 4). These results indicate that P2X1 receptor activation leads to dynamin dependent receptor internalisation. When dynasore blocks this process there is reduced FRAP because of a combination of reduced removal of photo-bleached receptor from the cell surface and reduced receptor recycling.

### Does blocking receptor trafficking regulate P2X1 receptor currents?

**Fig. 5:**
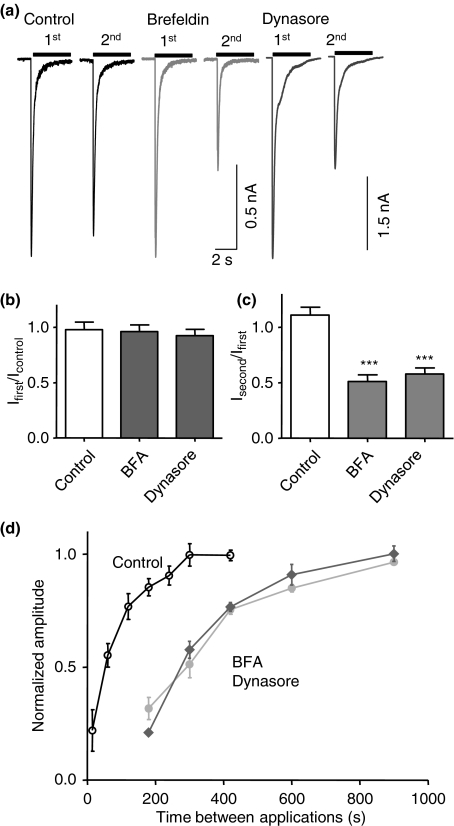
Brefeldin A and dynasore sensitive recovery of P2X1 receptor currents from desensitisation. (a) Representative currents evoked by first and second applications of α,β-meATP (10 μM) with a time interval of 5 min recorded in the pemeabilised patch whole-cell configuration in non-treated (control) cells, cells treated with BFA (10 μg/mL for 2 h) or dynasore (80 μM for 1 h). (b) BFA or dynasore had no effect no effect on the amplitude of the first responses (*n* = 7–10). (c) Recovery from desensitisation (peak current amplitude Isecond/Ifirst) with a 5-min interval was reduced following BFA or dynasore treatment (*n* = 5–8). (d) Time-course of recovery from desensitization of P2X receptor currents for control, BFA and dynasore-treated cells expressed as fraction of the initial response to α,β-meATP (10 μM, *n* = 8–12 for each point). ***p < 0.001.

The FRAP studies indicate that the P2X1 receptor shows both constitutive and agonist induced trafficking however they do not determine the contribution of trafficking to responsiveness of the receptor. To address this we have used patch clamp recording of P2X1 receptor currents. BFA and dynasore treatment had no effect on the initial response of the P2X1 receptor to α,β-meATP (Fig. 5) (mean current amplitudes of 257 ± 29, 206 ± 24 and 251 ± 53 pA/pF for control brefeldin and dynasore treatment respectively, *n* = 5–3). In the permeabilised patch configuration α,β-meATP evoked rapidly desensitising inward P2X1 receptor currents. These were reproducible when a 5-min interval was given between applications for recovery from desensitisation as reported previously (Lewis and Evans 2000). However BFA and dynasore produced a ∼ 50% decrease in the P2X1 receptor response to the second application of α,β-meATP applied 5 min later. Further studies varying the interval between α,β-meATP applications showed that both BFA and dynasore slowed the time-course of receptor recovery from desensitisation (time to 50% recovery 55 s, 300 s and 270 s for control, BFA and dynasore respectively). These results suggest that P2X1 receptor trafficking plays an important role in regulation of repeated P2X1 receptor mediated responses.

### GPCR/second messenger mediated regulation of P2X1 receptors

**Fig. 6:**
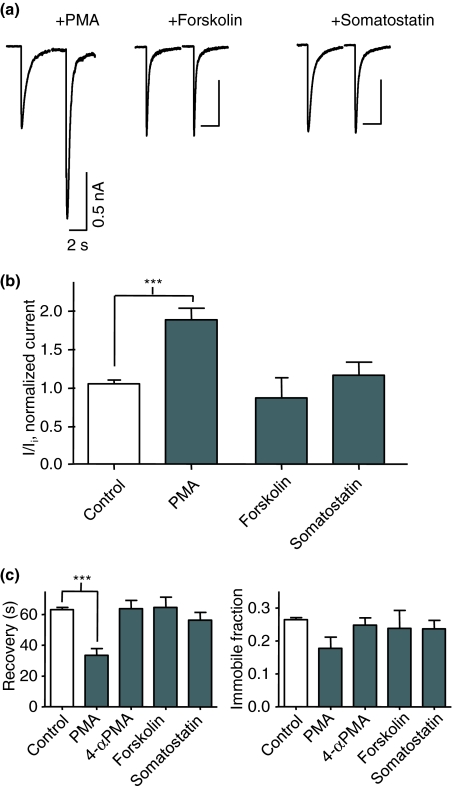
P2X1 receptor mediated currents and FRAP are regulated by PMA but not by cAMP dependent mechanisms. (a) Representative currents evoked by applications of α,β-meATP (10 μM) recorded in the permeabilised patch whole-cell configuration before and after addition of PMA (100 nM), forskolin (10 μM) or somatostatin (500 nM) to extracellular solution. (b) Summary data of the effects of PMA, forskolin and somatostatin on P2X1 receptor mediated currents expressed as fraction of the initial response to α,β-meATP (10 μM, *n* = 4–8). (c) FRAP time constants and immobile fractions for cells treated with PMA, 4-αPMA, forskolin or somatostatin (*n* = 5–8). PMA only increase the rate of recovery after photo-bleaching (33.43 ± 4.33, *n* = 5, *p* < 0.0001). Immobile fractions were not altered. ***p < 0.001.

P2X1 receptor responses can be potentiated by stimulation of Gαq coupled mGlur1α and P2Y receptors, this effect can be mimicked by application of the phorbol ester PMA (Vial *et al.* 2004; Ase *et al.* 2005) and involves amino acids in the intracellular amino terminus (Wen and Evans 2009). However whether this has an effect on receptor mobility/trafficking or whether Gαi or Gαs -protein coupled receptors and signalling pathways can regulate P2X1 receptors is unknown. We therefore determined the effects of PMA, forskolin to stimulate protein kinase A and mimic Gαs receptor activation, or stimulation of the endogenous HEK cell Gαi coupled somatostatin receptor on P2X1 receptor mediated currents and FRAP. PMA (100 nM) potentiated P2X1 receptor currents by 104.7 ± 17.8% (Fig. 6). Forskolin (10 μM) or somatostatin (500 nM) had no significant effect on P2X1 receptor currents or on receptor FRAP (Fig. 6). These results indicate that P2X1 receptors can be regulated by Gαq coupled, but not Gα1 and Gαs coupled GPCRs. Interestingly PMA treatment increased the FRAP recovery rate with no effect on the immobile fraction (the inactive phorbol ester 4-α-PMA, also 100 nM, had no effect on recovery rate or immobile fraction) (Fig. 6) suggesting that an effect on receptor trafficking contributes to the P2X1 receptor potentiation.

## Discussion

Fluorescence recovery after photo-bleaching provides a powerful technique to look at receptor movement in real time. This approach, along with parallel electrophysiological studies on P2X1 receptor currents, has allowed us to investigate the contribution of mobility and trafficking to P2X1 receptor function. These studies show that the P2X1 receptor is not static on the cell surface but undergoes considerable mobility and turnover. Following photo-bleaching, recovery of fluorescence can result from the lateral diffusion of receptors adjacent to the bleach site, as well as trafficking of receptors to the cell surface. If the fluorescence does not fully recover this indicates that some of the bleached receptors remain as an immobile fraction. P2X1-eGFP receptor fluorescence recovered with a time constant of ∼ 60 s and the immobile fraction accounted for ∼ 25% of the receptors expressed at the surface. These results indicate that in the 5-min period there is ∼ 75% dynamic movement of the receptors at the cell surface in the bleached area. The FRAP profile of the P2X2 receptor (faster recovery and smaller immobile fraction) shows greater mobility in the membrane. This is interesting as the P2X2 receptor has a longer C-terminal tail and as a larger protein one might expect it to move more slowly in the membrane. Therefore, these results suggest that there are subtype dependent differences in mobility of the P2X receptors. We have previously suggested that the P2X1 receptor may be regulated by interacting proteins (Vial *et al.* 2004; Wen and Evans 2009) and the reduced speed of mobility and increased immobile fraction may reflect this.

In this study, we have characterised the contribution of new and recycling P2X1 receptors to the mobile fraction. The modest effects of the protein synthesis inhibitor cycloheximide on FRAP suggest that insertion of newly synthesised P2X1 receptors at the cell surface makes only a small contribution to recovery from photo-bleaching. This is consistent with a recent report on the cell surface expression of P2X1 receptors (Vacca *et al.* 2009). It appears that the importance of newly synthesized receptor is subtype dependent as P2X7 receptors (Gu and Wiley 2006) also make a limited contribution to receptor turnover whereas they make a significant contribution to P2X3 receptor expression (Vacca *et al.* 2009). BFA treatment leads to the collapse of the Golgi and its redistribution to the endoplasmic reticulum blocking transit of new proteins to the cell surface (Chardin and McCormick 1999) and has similar effects to cycloheximide. BFA in addition blocks other vesicular transport processes (van Dam *et al.* 2002; Butterworth *et al.* 2005; Kim and Ryan 2009). Two lines of evidence suggest that it is this latter action of BFA, on vesicular transport, that accounts for the decreased recovery from FRAP (increased immobile fraction) for the P2X1 receptor. First, the lack of effect of cycloheximide on recovery (this study) or surface expression (Vacca *et al.* 2009) suggests that new protein synthesis does not play a major role. Second, if BFA was acting to block the insertion of new receptors treatment with α,β-meATP would not rescue the response as new receptors are not available for trafficking. These results suggest that P2X1 receptors are constitutively internalised and recycled by a BFA sensitive pathway. P2X3 and P2X4 receptors also show constitutive internalisation. This involves ubiquitination of P2X3 receptors (Vacca *et al.* 2009) and for P2X4 receptors interaction of a non-canonical motif in the C-terminus that binds the adaptor protein AP2 (Royle *et al.* 2005) is involved and a dileucine-like motif in the N-terminus can target the receptors to lysosomes (Bobanovic *et al.* 2002). These motifs are absent from the P2X1 receptor and the molecular mechanism underlying BFA sensitivity remains to be determined. Our results showing the lack of effect of BFA on P2X2 receptor FRAP support previous studies that P2X2 receptors are not subject to constitutive internalisation (Bobanovic *et al.* 2002) and demonstrate that BFA does not have a generalised effect on receptor trafficking. Taken together these results demonstrate significant differences in trafficking between P2X receptor family members.

Activation of the P2X1 receptor with α,β-meATP increased the rate of recovery from photo-bleaching in a manner similar to that reported for ATP on P2X2 receptor mobility (Chaumont *et al.* 2008). We now show that calcium influx through the P2X receptor, rather than receptor activation *per se*, is mainly responsible for this effect as the accelerated recovery was mimicked by raising the intracellular calcium concentration with thapsigargin in the absence of P2X receptor stimulation. The ability of BAPTA to block the speeding of recovery by thapsigargin but not by α,β-meATP further suggests that it is likely to be a localised effect of a calcium microdomain close to the cell surface. These results therefore show that P2X receptor activation can lead to an increase in receptor mobility.

P2X1 receptor activation results in receptor internalisation and recycling (Dutton *et al.* 2000; Li *et al.* 2000; Ennion and Evans 2001). Dynasore treatment had no effect on P2X1 receptor FRAP in the absence of stimulation. However the reduction by dynasore of the level of recovery (increased immobile fraction) following α,β-meATP treatment suggests that receptor internalisation and recycling were reduced and bleached P2X1 receptors could not be replaced at the cell surface. This reduction in recovery in activated P2X1 receptor FRAP (but not at rest) by dynasore suggests that the agonist stimulated internalization/recycling of the receptor is dynamin/clathrin dependent. The lack of observation of internalised receptor following agonist stimulation demonstrates that the receptor is rapidly trafficked back to the cell surface consistent with previous biochemical studies (Ennion and Evans 2001).

The different effects of BFA and dynasore on FRAP suggest that these drugs regulate different aspects of P2X1 receptor trafficking. This is consistent with the opposite effects of α,β-meATP treatment in response to brefeldin or dynasore; α,β-meATP treatment reversed the increase in immobile fraction induced by brefeldin and increased the immobile fraction following dynasore treatment. For brefeldin this may result from agonist induced trafficking of the receptor. For dynasore the increase in immobile fraction following receptor stimulation suggests that agonist induced trafficking plays an important role in the cell surface expression of P2X1 receptors.

Our electrophysiological studies showed that both BFA and dynasore regulate recovery of P2X1 receptor currents from desensitisation. Inhibition of trafficking by either BFA or dynasore reduced the response to a second application of agonist at 5 min by ∼ 50% and increased the time required for P2X1 receptors to recover from desensitisation. These results suggest that internalisation and recycling to the cell surface plays an important role in recovery from desensitisation. One possibility is that the acidic environment that the extracellular ligand binding domain of the receptor is exposed to in the endosome could facilitate the dissociation of agonist from the receptor as has been suggested for other receptors (Maxfield and McGraw 2004).

The electrophysiological studies also reveal how FRAP may underestimate the importance of trafficking in recovery from desensitisation as there was little or no effect on FRAP with brefeldin or dynasore between the first and second photo-bleach, however P2X1 receptor currents were reduced by ∼ 50% in electrophysiological studies. These discrepancies can be explained by two factors (i) the small area photo-bleached and (ii) the FRAP technique looks at recovery of fluorescence and not recovery of receptors from a desensitised state. In FRAP only the area of interest was photo-bleached. The pool of adjacent unbleached receptors at the surface available for lateral diffusion allows initially for significant recovery after the first photo-bleach but then on subsequent bleaching the pool becomes depleted and reduced trafficking of new receptors becomes apparent as a decrease in the level of recovery. The electrophysiological studies measure the functional activity of all the P2X receptors on the cell, whereas the fluorescence gives a measure of the number of receptors but not whether they are in an available or desensitised state. Therefore in FRAP studies the fluorescence recovery may correspond to movement of desensitised receptors into photo-bleached area. For example with BFA treatment the recovery of the immobile fraction with α,β-meATP (that was applied to the whole cell) may result from increased lateral diffusion of fluorescent, but desensitised receptors, from an adjacent area consistent with the increased mobility of the receptors following receptor activation and calcium influx.

The FRAP and electrophysiological studies suggest that there are two trafficking pathways for the P2X1 receptor; a constitutive BFA sensitive one and a receptor-stimulated dynasore-sensitive one. Support for two pharmacologically distinct pathways comes from a recent study showing that dynasore dependent internalization of the TRPV5 (transient receptor potential cation channel, subfamily V, member 5) calcium channel is blocked by dynasore but not by BFA (van de Graaf *et al.* 2008). The electrophysiological studies clearly show that trafficking can regulate P2X1 receptor responsiveness. On the other hand, as a positive feedback, trafficking events can also be regulated by activation of P2X1 receptor. The increase in time constant of recovery of P2X1 FRAP following phorbol ester treatment is consistent with a decrease in the time required for recovery from desensitisation (Ase *et al.* 2005). Overall these studies highlight that trafficking plays an important role in the control of the functional expression of P2X1 receptors and their regulation.

## Abbreviations used:

α,β-meATP: α,β-methylene ATP
BAPTA-AM: 1,2-Bis(2-aminophenoxy)ethane-N,N,N′,N′-tetraacetic acid tetrakis(acetoxymethyl ester)
BFA: brefeldin A
eGFP: enhanced green fluorescent protein
FRAP: fluorescence recovery after photo-bleaching
GPCRs: G protein coupled receptors
